# Centenarians and diet: what they eat in the Western part of Sicily

**DOI:** 10.1186/1742-4933-9-10

**Published:** 2012-04-23

**Authors:** Sonya Vasto, Claudia Rizzo, Calogero Caruso

**Affiliations:** 1 Department of Molecular and Biomolecular Sciences (STEMBIO), University of Palermo, Via Archifari 32, 90213, Palermo, Italy; 2 Department of Pathobiology and Medical and Forensic Biotechnologies (DIBIMEF), University of Palermo, Corso Tukory, 211, 90138, Palermo, Italy; 3 Immunohaemathology Unit, University Hospital, University of Palermo, Via del Vespro, 127, 90137, Palermo, Italy

**Keywords:** Ageing, Centenarian, Longevity, Mediterranean diet

## Abstract

This paper pays attention to the modifiable lifestyle factors such as diet and nutrition that might influence life extension and successful ageing. Previous data reported that in Sicily, the biggest Mediterranean island, there are some places where there is a high frequency of male centenarians with respect to the Italian average. The present data show that in Sicani Mountain zone there are more centenarians with respect to the Italian average. In fact, in five villages of Sicani Mountains, there were 19 people with an age range of 100–107 years old from a total population of 18,328 inhabitants. So, the centenarian number was 4.32-fold higher than the national average (10.37 vs. 2.4/10,000); the female/male ratio was 1.1:1 in the study area, while the national ratio is 4.54:1. Unequivocally, their nutritional assessment showed a high adherence to the Mediterranean nutritional profile with low glycemic index food consumed. To reach successful ageing it is advisable to follow a diet with low quantity of saturated fat and high amount of fruits and vegetables rich in phytochemicals.

## Introduction

The Mediterranean diet has been widely recommended for a healthy lifestyle since Ancel Keys first used the term in 1975 [1]. The essential concept is that this is not a set of changes to our usual diet dictated by scientific experiments, but a set of food habits and recipes traditionally enjoyed by the ordinary people of Mediterranean countries, who have been found to have lower rates of coronary and other age-related chronic diseases, including cancer, than most developed countries [2,3].

In recent years, researchers have been extremely interested in the clear advantage of the Mediterranean nutritional recommendation that exists and is used in the Mediterranean surrounding area. In fact, there is no single Mediterranean diet but several interpretations based on the Mediterranean country’s political, economic and cultural tradition [4].

Since 1990, increasing evidence suggests that these diets have a beneficial influence on several diseases such as cardiovascular diseases, metabolic syndromes, hence showing protective effect on health and longevity [5-7]. Mediterranean diet is characterized by a high intake of monounsaturated fat, plant proteins, whole grains (fish is not always present), moderate intake of alcohol, and low consumption of red meat, refined grains, and sweets. Further, the consumption of large amount of olive oil and olives in meals dominates all the Mediterranean cuisine [8].

Historically, the beneficial properties of virgin olive oil were attributed to the high proportion of monounsaturated fatty acids (MUFAs), namely oleic acid, rather than to the phenolic fraction. Nevertheless, several seed oils, including sunflower, soybean, and rapeseed, rich in MUFA have been demonstrated to be ineffective in beneficially altering chronic disease risk factors. Therefore, it is likely that the polyphenols in olive oil may mediate these health benefits [8,9].

There are at least thirty-six structurally distinct phenolics that have been identified in virgin olive oil, but not all phenolic compounds and their concentration are present in every virgin olive oil. Such differences in the phenolic compound are dependent on several factors like the variety of the olive fruit, the region in which the olive fruit is grown, the agricultural techniques used, the maturity of the olive fruit at harvest, the extraction process and the storage method [10].

The Sicanian Mountains (or Sicani), bordered by Ficuzza wood in the North, Caltanissetta in the East, Salemi in the West and Agrigento to the South represent a very peculiar area where there is a high frequency of centenarians with respect to the Italian average [11,12]. The goal of this study was to characterize the dietary habits of centenarians residing around the Sicani mountains, in 5 villages, namely Giuliana, Bisacquino, Castronovo, Chiusa Scalafani, and Prizzi.

## Materials and Methods

19 centenarians (10 females and 9 males) living at home in the five municipalities of Bisacquino, Castronovo, Chiusa Scalafani, Giuliana and Prizzi in the Western part of Sicily, Italy, were identified for the present study. These villages are located above sea-level, on the South-Western edge of the Sicani Mountains. Subjects were individuated by general practitioners and their age checked in the birth registries. As a further control, in the interview, particular attention was paid to the concordance between reported age and personal chronologies (age of marriage and of military service for men, age of first and last pregnancy for women, age of children, among others). The subjects underwent a physical examination and a morning fasting blood venous sample was obtained for studying blood chemistry parameters. Anthropometric measures included height, weight, and the body mass index (BMI) [weight (kg)/height (m2)]. Furthermore, the Mini Nutritional Assessment (MNA), Basic Activities of Daily Living (ADL) and the Instrumental Activities of Daily Living (IADL) were administrated. ADL and IADL were assessed by interviewing participants and their caregivers [13,14]. Physical items (meal taking, bowel and bladder continence, standing ability, extent of general activities, bathing and dressing abilities), sensory items (auditory acuity and eyesight) and cognitive abilities (comprehension and self-expression) were included in the ADL. Each item was classified into five categories of self-sufficiency: completely independent, independent but slow, independent with difficulty, partially dependent and completely dependent, using a point score from 12 to 1, respectively.

The study was approved by local University Hospital Ethics Committee; the purpose and procedures of the study were explained to the subjects, and informed and written consent was obtained from the participants or caregivers.

## Results

Table 1 depicts the prevalence of centenarians in Italy and in the study area; we have identified 19 centenarian, 10 female and 9 males among a population of 18,327 inhabitants. In this area the centenarian number was 4.32-fold higher than the national average (10.37 vs. 2.4/10,000). It is noteworthy that the male centenarian number was 11.51-fold higher than the national average (10.24 vs. 0.89/10,000). Female/male ratio was 1.1:1 in the study area, while the national ratio was 4.54:1.

**Table 1:** Distribution of Centenarian population in five villages of Sicani Mountains and in Italy

	**Total Population**	**Males**	**Females**	**Total Centenarians**	**Male Centenarians**	**Female Centenarians**
Sicani Mountains	18,328	8,793	9,535	19	9	10
	(10.37)	(10.24)	(10.48)			
Italy	60,626,442	29,413,274	31,213,168	14,473	2,612	11,861
	(2.39)	(0.89)	(3.80)			

Centenarian prevalence x 10,000 inhabitants between brackets. For data on Italian Centenarians see [15].

All the centenarians live in a family home, mostly with their relatives. Individual ADL and IADL scores were in the category of moderately independent for both genders. A good anamnesis on a single individual reported a poor auditory acuity and poor eyesight, while they were free from cardiac heart disease, severe cognitive impairment, severe physical impairment, clinically evident cancer or renal insufficiency.

**Figure 1:**
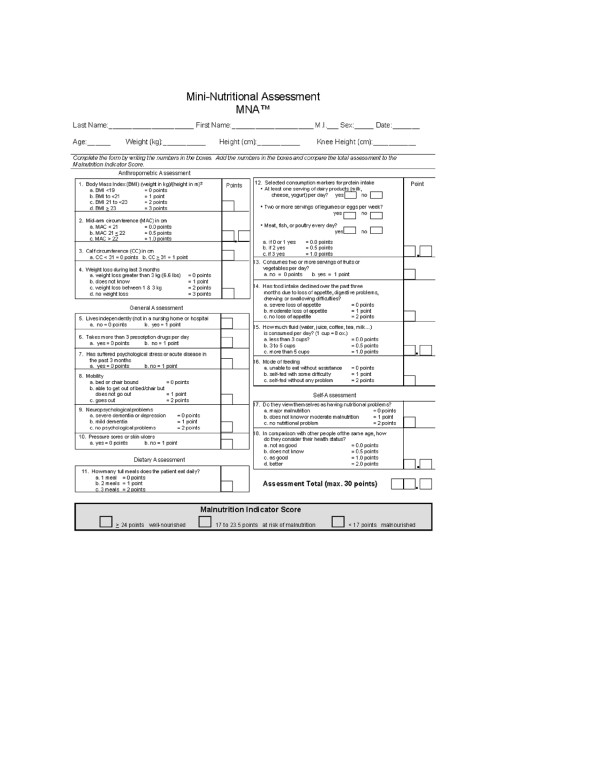
Example of MNA administered to healthy centenarians.

**Figure 2:**
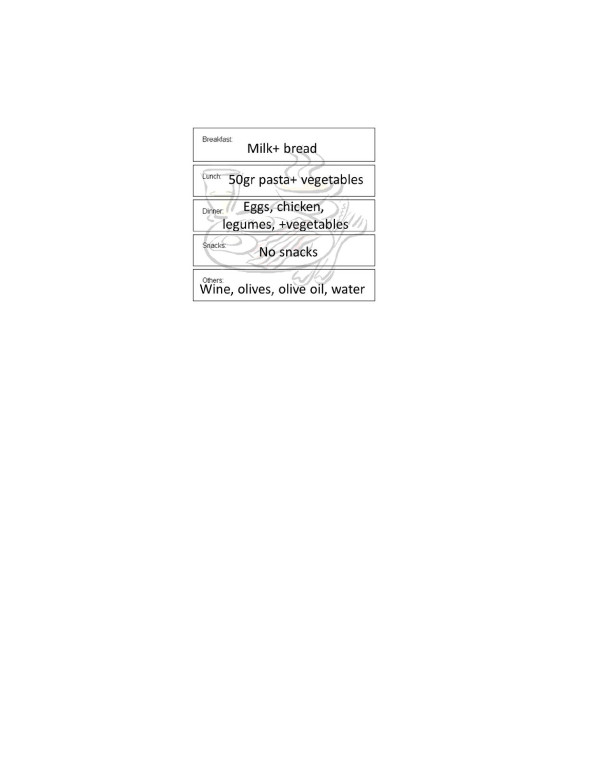
Example of daily menu.

**Figure 3:**
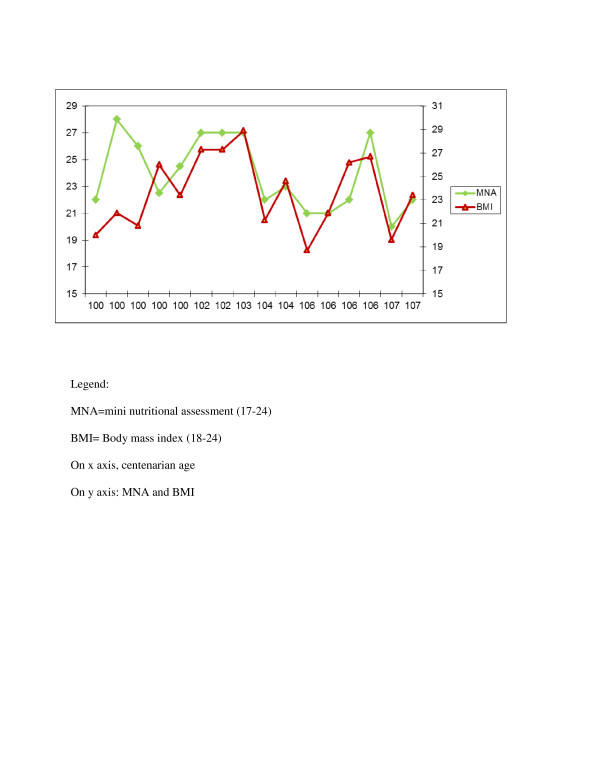
Mini nutritional assessment and Body mass index in healthy centenarian population.

In Figure 1, MNA example administrated to healthy centenarian is shown [13], whereas Figure 2 reports a typical daily diet. Centenarians recruited in these area tended to be physically active, non-obese, small in stature, with a regular BMI (23.6 ± 3.1), suggestive of some degree of calorie restriction with high intake of seasonal plant food and low meat intake. Their diet shows a low glycemic index because low of refined carbohydrate (no white bread, low amount of pasta, no sweeteners, sweet beverages, can food, frozen already prepared vegetables or dishes, cookies cakes or snacks). Furthermore, they have a good intake of olive and virgin olive oil from different cultivar namely: Nocellara of Belice, Biancolilla, Giarraffa and Ogliarola that seems to have important anti-oxidant properties (unpublished data). In Figure 3, BMI and MNA are plotted together showing a perfect accordance between nutrition and body mass index.

## Discussion

In Italy in 2010 the population aged one hundred years and over has 14 thousand units, hence the centenarian prevalence is 2.4/10.000. Currently there are almost five centenarian women for one man. However, in Italy there is a North to South gradient in the female/male ratio in centenarians [15]. To gain insight into the role of gender and environment, we have started a demographic study in Sicily showing that in mountain zones of Sicily there is a zone of male longevity with similar features to those found in Sardinia, in the so-called Blue Zone [11,12,16]. In both cases the municipalities concerned do not include polluted areas and are small, with the lowest number of inhabitants. Therefore, longevity is more prevalent in men living in a small town, without pollution, likely because of different working conditions, different life style *i.e*. reduced smoking and alcohol abuse and Mediterranean diet. Accordingly, both these areas in Sicily and in Sardinia also share low mortality from cancers and cardiovascular diseases [11,16-18].

One of the places in the Western part of Sicily, characterized by a high presence of oldest old people, is the area of “Monti Sicani”, between the provinces of Agrigento and Palermo [11,12]. “Monti Sicani” encompasses the area between the cities of Palermo and Agrigento from North to South and between the city of Caltanissetta and Trapani from West to East. The territory is characterized by a hilly area of clayey sandstone or pasture and a mountain area above 900 m, consists of pelagic limestone rocks of the Mesozoic. This area is characterized by olive tree agriculture, which tolerates a large range of soil conditions, preferring a neutral to alkaline soil type.

Looking at the national ratio of centenarian per inhabitants in these area we have found more than a four-fold increase in centenarian, and regarding male female ratio of 1.1:1 times. Since Sicilian population genetics structure is very homogeneous and in Hardy-Weinberg equilibrium [19], the explanation for these data probably resides in the environmental characteristics of the study sample.

In this area, we have found a high number of centenarians in good health, with a notable increase of male centenarians. Unequivocally, their nutritional assessment showed a high adherence to the Mediterranean nutritional profile with low glycemic index food consumed. According to the scores of ADL and IADL, centenarians of both gender demonstrated a good level of independency. They did not have any cardiac risk factors or major age related diseases (e.g. cardiac heart disease, severe cognitive impairment, severe physical impairment, clinically evident cancer or renal insufficiency), although some had decreased auditory and visual acuity. Their life is characterized by social networking, acceptable physical activity and small amount of food divided among three meals, which contain a little amount of carbohydrate and meat and a lot of seasonal fruit and vegetables. In relation to biochemical parameters in centenarians, most biochemical parameters including cholesterol and triglycerides were within normal limits (data not shown) and better than those previously reported in a study of Sicilian elderly [20]. Furthermore, this reported modified Mediterranean-style show a low glycemic load.

The Glycemic index (GI) is defined as a kinetic parameter that reflects the potency of food to raise blood glucose level and glucose clearance. The GI of a specific diet is calculated by averaging the GI values of the food items, statistically weighted by the carbohydrate contribution. Diets based on refined carbohydrate foods that are quickly digested, absorbed, and metabolized (i.e., high glycemic index diets) have been associated with increased risk of lifestyle diseases in particular with an increased risk of type 2 diabetes, because of postprandial hyperglycemia and hyperinsulinemia related to eating high-GI carbohydrates. Low GI is known to protect against heart disease in women, and cross-sectional studies indicate low GI may reduce high-density-lipoprotein cholesterol and triacylglycerol levels in both sexes. More interesting, new observational studies have reported increased risks of coronary heart disease associated with higher intakes of carbohydrates from high glycemic index foods. Epidemiological evidence has emerged linking dietary glycemic index to visceral fat and inflammatory disease mortality [21-23]. Therefore, the Mediterranean diet is an anti-inflammatory diet [24]: it is puzzling that Italian centenarians are remarkably enriched in “good” genotypes involved in control of inflammation, confirming that a good control of inflammatory responses (genetic and/or environmental) is advantageous for longevity [25,26].

Overall, our data confirm our previous suggestion that longevity concerns subjects, living in small town, without pollution, with different working conditions, lifestyles and close adherence to a Mediterranean diet. The reason why longevity has been observed particularly in small municipalities is not surprising. It is a well established, in fact, that individuals with greater access to social support and family network have better health and lower levels of mortality, particularly when adult daughters are present. Nevertheless, our data are collected in a relative small sample of subjects; accordingly, our data needed to be confirmed by larger population-based studies.

To conclude, our work show a segment of our population that is growing faster and represent a typical example of successful ageing. Genetic and environment play a major role in healthy ageing and nutrition has a significant influence. It has been estimated that the number of centenarians will approach 3.2 million world-wide by 2050 and that means an 18-fold increase with respect to the last century [27]. Consequently, understanding the influence of dietary life-style in the process of healthy ageing is of paramount importance to development new strategies leading to healthy life extension. Finally, our results are consistent with data described in Dan Buettner’s book on the importance of the diets in 5 populations with high longevity [28]. To reach successful ageing it is advisable to follow a diet with low quantity of saturated fat and high amount of fruits and vegetable, rich in phytochemicals.

## Competing interests

The authors declare that they have no competing interests.

## Authors’ contributions

SV wrote the paper. All authors edited the paper and approved its final version.
